# Feasibility and clinical utility of local rapid Nanopore influenza A virus whole genome sequencing for integrated outbreak management, genotypic resistance detection and timely surveillance

**DOI:** 10.1099/mgen.0.001083

**Published:** 2023-08-17

**Authors:** Tom G. S. Williams, Luke B. Snell, Christopher Alder, Themoula Charalampous, Adela Alcolea-Medina, Jasveen K. Sehmi, Noor Al-Yaakoubi, Gul Humayun, Shahjahan Miah, Angie Lackenby, Maria Zambon, Rahul Batra, Sam Douthwaite, Jonathan D. Edgeworth, Gaia Nebbia

**Affiliations:** ^1^​ Department of Infection, Guy’s & St. Thomas’ NHS Foundation Trust, London, UK; ^2^​ Centre for Clinical Diagnostics & Infectious Disease Research, Guy’s & St. Thomas’ NHS Foundation Trust, London, UK; ^3^​ Department of Infectious Diseases, King’s College London, London, UK; ^4^​ Infection Sciences, Synnovis, London, UK; ^5^​ United Kingdom Health Security Agency (UKHSA), London, UK

**Keywords:** influenza A, whole genome sequencing, nosocomial outbreaks, Nanopore, antiviral drug resistance, avian influenza

## Abstract

Rapid respiratory viral whole genome sequencing (WGS) in a clinical setting can inform real-time outbreak and patient treatment decisions, but the feasibility and clinical utility of influenza A virus (IAV) WGS using Nanopore technology has not been demonstrated. A 24 h turnaround Nanopore IAV WGS protocol was performed on 128 reverse transcriptase PCR IAV-positive nasopharyngeal samples taken over seven weeks of the 2022–2023 winter influenza season, including 25 from patients with nosocomial IAV infections and 102 from patients attending the Emergency Department. WGS results were reviewed collectively alongside clinical details for interpretation and reported to clinical teams. All eight segments of the IAV genome were recovered for 97/128 samples (75.8 %) and the haemagglutinin gene for 117/128 samples (91.4 %). Infection prevention and control identified nosocomial IAV infections in 19 patients across five wards. IAV WGS revealed two separate clusters on one ward and excluded transmission across different wards with contemporaneous outbreaks. IAV WGS also identified neuraminidase inhibitor resistance in a persistently infected patient and excluded avian influenza in a sample taken from an immunosuppressed patient with a history of travel to Singapore which had failed PCR subtyping. Accurate IAV genomes can be generated in 24 h using a Nanopore protocol accessible to any laboratory with SARS-CoV-2 Nanopore sequencing capacity. In addition to replicating reference laboratory surveillance results, IAV WGS can identify antiviral resistance and exclude avian influenza. IAV WGS also informs management of nosocomial outbreaks, though molecular and clinical epidemiology were concordant in this study, limiting the impact on decision-making.

## Data Summary

Read data are available on the Sequence Read Archive (BioProject: PRJNA930694). Consensus genomes used for analysis are available on GenBank (accession numbers OQ763889–OQ764790), except for the polymerase acidic protein (PA) segments for sample ED61 and ED49 which failed GenBank QC checks and are instead provided in Table S5, available in the online version of this article, alongside their error flags. United Kingdom Health Security Agency (UKHSA) reference laboratory sequences are available on GISAID, with the GISAID EpiFlu names for sequenced samples listed in Table S3.

**Impact Statement**

Here we apply recent advances in Nanopore sequencing to demonstrate the feasibility and utility of rapid local influenza A virus (IAV) whole genome sequencing (WGS), which until now has been typically performed by reference laboratories using Illumina platforms. Using a Nanopore protocol, IAV genomes could be generated in 24 h and be provided to clinicians in a clinically relevant timeframe. We demonstrate that these IAV WGS results can be used to inform management of nosocomial outbreaks, guide treatment decisions by identifying antiviral resistance, exclude avian influenza and provide timely genomic surveillance data. The protocol is accessible to institutions with Nanopore platforms acquired during the SARS-CoV-2 pandemic, and therefore distribution of IAV WGS more widely throughout laboratory networks is now feasible.

## Introduction

After two years with low case numbers, influenza A virus (IAV) has returned as a dominant winter respiratory virus in England, becoming the most frequently detected respiratory virus at sentinel surveillance sites in December 2022 [1]. Nosocomial IAV infection is associated with significant morbidity and mortality [2] and is therefore an infection prevention and control (IPC) priority.

Since the onset of the SARS-CoV-2 pandemic, locally performed whole genome sequencing (WGS) with rapid turnaround times has been demonstrated to impact hospital outbreak management [3], guide individual treatment decisions [4] and provide timely surveillance [5]. Nosocomial IAV outbreaks have also been successfully investigated by WGS using Illumina platforms [6–8]. Recent advances in Nanopore IAV WGS, which have been applied to swine and avian samples [9, 10], are yet to be applied in a clinical setting.

Here we report on the feasibility of using rapid Nanopore sequencing to inform the management of nosocomial IAV outbreaks in two tertiary hospitals. Adapting previously published methods to reduce hands-on time, we demonstrate the utility of a 24 h workflow to generate a consensus sequence from an RNA extract. We also demonstrate the potential of local sequencing to guide treatment decisions for individual patients and provide timely surveillance for determining prevalent IAV clades, monitoring community antiviral resistance rates and rapidly excluding avian influenza when samples fail PCR subtyping.

## Methods

### Setting, routine testing, WGS sample identification and clinical-laboratory liaison

The study was conducted at Guy’s and St Thomas’ NHS Foundation Trust, a secondary care provider and tertiary referral centre with two hospital sites in London, UK. Inpatients with suspected viral respiratory tract infection had routine nasopharyngeal swabs collected for analysis using the AusDiagnostics Universal 24-well Respiratory Pathogen Panel, a nested PCR assay, to test nucleic acids extracted from samples using a QIAsymphony (Qiagen) according to the manufacturer’s instructions. Symptomatic patients presenting to the Emergency Department (ED) had point-of-care (POC) testing using the Liat (Roche) or Xpert Xpress (Cepheid) platforms. In the main text here, corrected Ct results are provided for samples tested on the AusDiagnostics Panel, with AusDiagnostics take-off values reported in Table S3. Between 1 December 2022 and 20 January 2023 surplus IAV-positive nucleic acid extracts post-AusDiagnostics testing were retrieved for WGS for all patients with suspected nosocomial IAV infection, with no corrected Ct value threshold. Between 01 December 2022 and 2 January 2023 all IAV-positive ED samples with a Ct <25 were retrieved and nucleic acids extracted using a MagNA Pure 24 (Roche) according to the manufacturer’s instructions, for parallel sequencing to link with any suspected nosocomial cases and provide a local genetic background of circulating community IAV. Sequencing was performed linked with timing of first reported nosocomial cases or further epidemiologically linked cases to provide results for consideration at subsequent IPC meetings. Collection of surplus samples and linked clinical data was approved by South Central—Hampshire B REC (20/SC/0310).

### WGS, consensus sequence generation, clade identification and drug resistance screening

Amplified cDNA was generated using a modified one-step reverse transcriptase PCR (RT-PCR) method developed for Nanopore sequencing platforms [11], which utilizes primers targeting the conserved packaging sequences at the end of each genome segment [12]. Modifications from the method published by King *et al*. [11] include the use of a second RT-PCR for each sample, using TUNI 12.4 and TUNI 13, and the omission of magnetic bead clean-up of individual samples prior to library preparation. The sequencing library was prepared with a rapid library preparation kit (SQK-RBK110.96) using a ×0.4 pooled barcoded sample volume to Ampure XP bead volume ratio and sequenced on an R9.4.1 flow cell using a GridION Mk1. On each sequencing run, in addition to clinical samples, a no-template control, H1N1 control (A/Puerto Rico/8/1934, Zeptometrix) and H3N2 control (A/Hong Kong/2671/19 Zeptometix) were sequenced to confirm the performance of the run, demonstrate between-run reproducibility, and identify any systematic insertions or deletions in consensus generation. A further non-IAV in-run control was introduced at the library preparation stage, with lambda phage DNA used to provide a measure of barcode misassignment. A full protocol is available at protocols.io (dx.doi.org/10.17504/protocols.io.n2bvj655wlk5/v1).

Consensus genome generation, clade identification and drug resistance screening were performed using an end-to-end in-house python script (https://github.com/GSTT-CIDR/influenza_r9.4.1_rbk_2022). Reads shorter than 500 bases were filtered, and adaptor sequences and barcodes were trimmed using NanoFilt 2.8.0 (https://github.com/wdecoster/nanofilt). Human reads were removed by mapping against GRCh38.p13 with minimap v2.24-r1122 [13]. Consensus sequences were then generated from reads with IRMA v1.0.3 [14], using a modified version of the FLU-minion config file, and the homopolymer at positions 131–137 in the N2 neuraminidase (NA) gene segment was masked. Details of consensus genome assembly, control replicates and comparison with reference laboratory Illumina sequences are described in the Supplementary Methods and Table S1. Consensus sequences were included in further analysis if a segment was covered at a depth of 20 for >90 % of the segment length and the sequence contained fewer than 10 % ambiguous bases.

Nextclade CLI v2.9.1 (https://clades.nextstrain.org) was used to identify the clade of each sample based on hemagglutinin (HA) gene sequences [15] against reference A/California/07/2009 for pdmH1N1 and A/Wisconsin/67/2005 for H3N2. Translated protein sequences for NA and PA were generated from consensus sequences using nextalign CLI v2.9.1 and were screened for mutations associated with reduced susceptibility to NA inhibitors and PA inhibitors, using the reference list maintained by the WHO global influenza programme (https://www.who.int/teams/global-influenza-programme/), using the 3 May 2022 update.

### Molecular epidemiological analysis

Molecular epidemiological analysis was performed using an end-to-end in-house python script (https://github.com/GSTT-CIDR/influenza_r9.4.1_rbk_2022). For each segment, all consensus sequences taken forward for analysis were aligned with MAFFT v7.475 (https://github.com/GSLBiotech/mafft) [16], using the mafft-linsi command, alongside the Northern Hemisphere Winter Season 2022/2023 vaccine strains A/Victoria/2570/2019 (EPI_ISL_417210) and A/Wisconsin/588/2019 (EPI_ISL_404460) for pdmH1N1, and A/Darwin/9/2021 (EPI_ISL_2233240) and A/Darwin/6/2021 (EPI_ISL_2233238) for H3N2. For samples with consensus sequences recovered for all eight segments, a whole genome sequence was generated by concatenating aligned segments. Due to poor coverage of the polymerase basic protein 1 (PB1) gene segment in higher Ct value samples, a known limitation of the one-step RT-PCR [10, 17, 18], a second analysis was performed on a majority genome comprising concatenated aligned polymerase basic protein 2 (PB2), PA, HA, nucleoprotein (NP), NA, matrix (M) and non-structural (NS) gene segments. A third analysis was performed on aligned HA gene sequences alone.

For each of these three alignments (whole genome, majority genome and HA), pairwise SNP distances were calculated with snp-dists v0.7.0 (https://github.com/tseemann/snp-dists), and a maximum-likelihood phylogenetic tree was generated with iqtree v1.3.11.1 [19] using ModelFinder [20] and 1000 ultrafast bootstraps [21]. To allow manual screening for reassortment, maximum-likelihood phylogenetic trees were also generated for each segment individually. Trees were visualized with Toytree v1.0 (https://github.com/eaton-lab/toytree).

Samples were considered genetically linked if they differed by six or fewer SNPs across the whole genome and clustered together on the phylogeny with >95 % bootstrap support [6]. This threshold for genetic relatedness was derived in a previous study of a nosocomial IAV outbreak in London, UK. The study calculated cut-offs above which a pair of samples would not be genetically linked of 5.8–6.2 pairwise nucleotide differences for pairs of samples taken 0–7 days apart, using an estimate of substitution rates in London pdmH1N1 samples combined with sequencing error rates on the Illumina platform. We selected to use this threshold in our study given the similar geographical location, hospital setting and time between samples, using the assumption that Nanopore error rates would be equivalent or greater than Illumina error rates.

## Results

### Sample identification and whole genome recovery

**Fig. 1.**
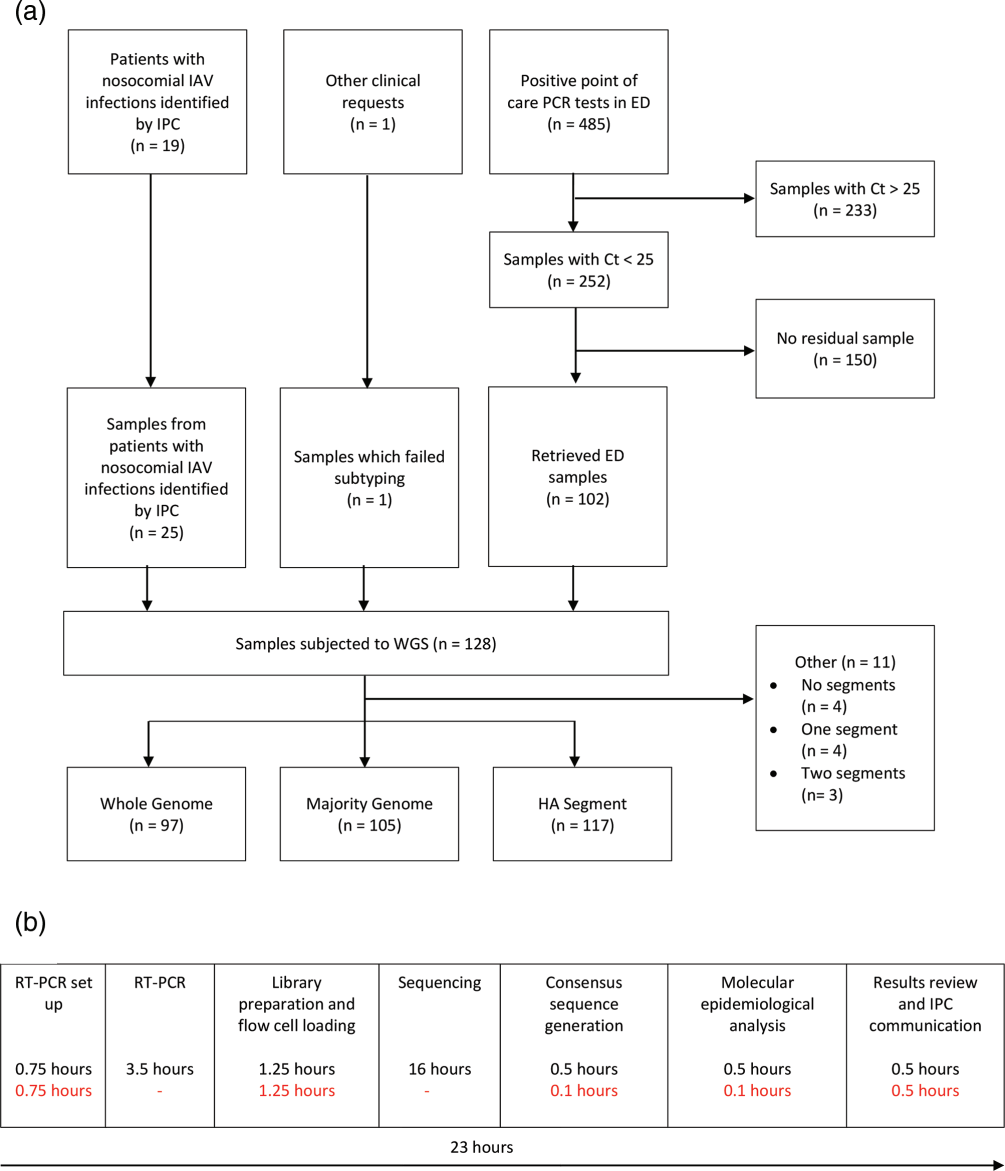
(**a**) IAV sample identification and categorization for molecular epidemiological analysis, and (**b**) workflow from RNA extraction to IPC communication of results – total time in black, hands-on time in red.

In total, 128 samples were retrieved for WGS from 122 patients (Fig. 1a). Twenty-five samples were from 19 patients with nosocomial IAV infection. There were 485 IAV-positive POC PCR tests taken in the Emergent Department (ED) between 1 December 2022 and 2 January 2023, of which 252 had a Ct <25 and 102 were recovered for WGS. A further sample from an immunosuppressed patient which had failed PCR subtyping was also sequenced at clinical request.

WGS was successful for 97/128 samples (75.8 %), a majority genome (all segments except PB1) was obtained for 105/128 samples (82.0 %), and the HA gene was obtained for 117/128 samples (91.4 %) (Fig. 1a). The number of samples with a consensus sequence for each segment is listed in Table S2 and read counts, mean coverage and number of ambiguous bases for each segment shown by sample are presented in Table S3.

IAV WGS was performed once a week. Accordingly, although the workflow can be completed within 24 h (Fig. 1b), the median turnaround time for outbreak samples from IPC request to reported result was four working days (interquartile range 4–7 days). Preliminary results based on pairwise SNP distances between all available sample sequences were reported to clinicians on the next working day after each sequencing run, with samples that differed by six or fewer SNPs considered likely to be linked.

### IAV outbreak investigation

Nineteen patients with nosocomial IAV infections and a contact link to another IAV case were identified by the IPC team. Cases were spread across five different wards (labelled A–E), with between two and five affected patients on each ward, and considered epidemiologically to represent five separate outbreaks (Table 1). In total, 18 of 19 patients with suspected nosocomial IAV infections first tested positive more than 48 h after admission, with 13 patients admitted for more than seven days before testing IAV positive (Table 1). The remaining patient (B3) first tested positive 36 h after admission following a previous negative swab and was identified as a suspected nosocomial acquisition by the IPC team. Twelve patients were male (63.2 %) and nine (47.4 %) were immunosuppressed. IAV PCR subtyping identified H1 in 11 patients across wards B, C and E, and H3 in the remaining eight patients on wards A and D.

**Table 1.** Clinical details and WGS results of patients with nosocomial IAV infections

Patient	Ward	Specialty	Age	Sex	Immunosuppressed	Admission date	First positive date	Inpatient days before positive	Type	Sample corrected Ct	Linked outbreak patients (WGS pairwise SNP distance)	Linked ED patients (WGS pairwise SNP distance)	Location at time of positive sample
A1	A	Renal	65	M	Renal transplant	17/11/2022	18/12/2022	31	H3	14.0	A5 (0), A3 (1)	None	Shared bay with A2, A3
A2	A	Renal	64	M	Renal transplant	23/11/2022	18/12/2022	25	H3	29.4	WGS fail	WGS fail	Shared bay with A1, A3
A3	A	Renal	67	M	Renal transplant	01/11/2022	19/12/2022	18	H3	18.3	A5 (1), A1.1 (1)	None	Shared bay with A1, A2
A4	A	Renal	20	F	ESRF on PD	04/12/2022	22/12/2022	18	H3	32.5	WGS fail	WGS fail	Side room
A5	A	Renal	52	F	Renal transplant	01/12/2022	22/12/2022	21	H3	15.8	A1.1(0), A3 (1)	None	Side room
B1	B	Vascular	53	M	No	09/11/2022	20/12/2022	11	H1	16.5	B2 (2)	ED96 (2)	Shared bay with B2
B2	B	Cardiology	55	M	No	16/12/2022	20/12/2022	4	H1	17.6	B1 (2)	ED96 (2)	Shared bay with B1
B3	B	Rheumatology	29	F	Takayasu’s arteritis on high-dose steroids	19/12/2022	21/12/2022	2	H1	23.2	B4 (0), B5 (0)	None	Shared bay with B4, B5
B4	B	Vascular	61	F	No	16/11/2022	25/12/2022	9	H1	14.0	B3.2(0), B5 (0)	None	Shared bay with B3, B5
B5	B	Vascular	75	F	No	22/11/2022	25/12/2022	33	H1	21.5	B3.2(0), B4 (0)	None	Shared bay with B3, B4
C1	C	Cardiology	37	F	No	16/12/2022	20/12/2022	4	H1	14.2	C4 (4), C3 (4)	ED88 (4)	Shared bay with C2
C2	C	Cardiology	84	F	No	10/12/2022	21/12/2022	11	H1	29.2	WGS fail	WGS fail	Shared bay with C1
C3	C	Cardiology	60	M	No	20/12/2022	24/12/2022	4	H1	24.5	C1 (4), C4 (2)	ED88 (2)	Shared bay with C4
C4	C	Vascular	83	M	No	21/12/2022	27/12/2022	6	H1	23.9	C1 (4), C3 (2)	ED88 (2)	Shared bay with C3
D1	D	Vascular	49	M	No	01/10/2022	29/12/2022	90	H3	15.6	D2 (1), D3 (1)	ED108 (1)	Different bay to D2, D3
D2	D	Vascular	76	M	No	20/11/2022	01/01/2022	42	H3	16.5	D1 (1), D3 (2)	ED108 (2)	Shared bay with D3
D3	D	Vascular	62	M	ESRF on HD	08/11/2022	02/01/2022	55	H3	17.1	D1 (1), D2 (2)	ED108 (2)	Shared bay with D2
E1	E	Haematology	66	M	Myeloma on chemotherapy	20/12/2022	31/12/2022	11	H1	14.6	E2 (2)	None	Side room
E2	E	Haematology	73	M	Hodgkin’s lymphoma	13/12/2022	01/01/2023	19	H1	17.0	E1 (2)	None	Side room

ED, Emergency Department; ESRF, end stage renal failure; HD, haemodialysis; PD, peritoneal dialysis.

**Fig. 2.**
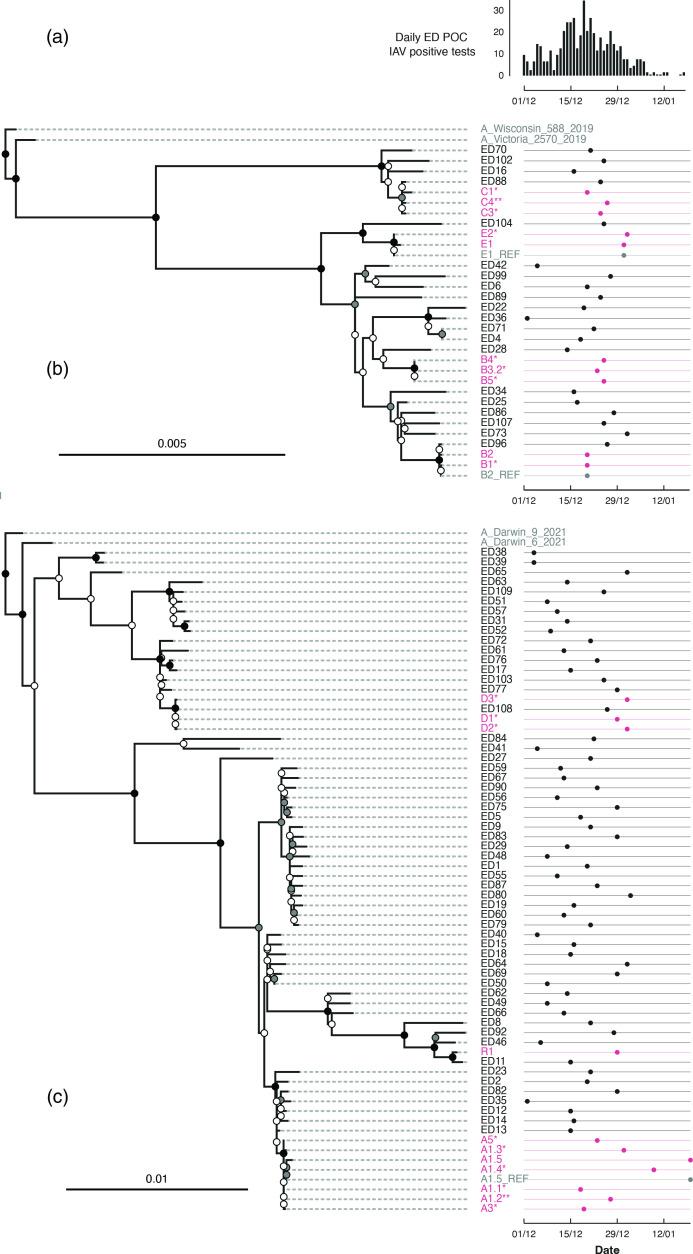
IAV incidence (**a**) and WGS maximum-likelihood phylogeny with sample dates of pdmH1N1 (**b**) and H3N2 (**c**) samples – alphanumeric names refer to patient location (e.g. A for ward A, ED for Emergency Department) followed by a patient number. Patients with multiple samples are labelled using a numerical suffix, for example A1.1 for the first sample and A1.2 for the second sample. *Samples with no SNP differences to a reference laboratory complete genome sequence; **samples with no SNP differences to a reference laboratory partial genome sequence. Samples from outbreak patients and clinical requests are highlighted in red. Reference laboratory sequences differing from local sequences are shown with the suffix ‘REF’. Node colour represents bootstrap support, with black nodes indicating 100 % support, grey 95–99 % support and white <95 % support. Outgroups are the Northern Hemisphere Winter Season 2022/2023 vaccine strains, A/Victoria/2570/2019 (EPI_ISL_417210) and A/Wisconsin/588/2019 (EPI_ISL_404460) for pdmH1N1, and A/Darwin/9/2021 (EPI_ISL_2233240) and A/Darwin/6/2021 (EPI_ISL_2233238) for H3N2. Maximum-likelihood phylogeny scale bars represent nucleotide substitutions per site. POC, point-of-care.

WGS was performed successfully on 16/19 (84.2 %) outbreak patients, including all patients with a sample with a corrected Ct <29. WGS grouped outbreak patients into six clusters using previously published criteria of six or fewer SNP differences and clustering together on the phylogeny with >95 % bootstrap support [6]. There was a maximum of four SNPs between any two patients within a cluster, with four SNPs between patient C1 and patients C3 and C4 (Table 1). Patients within all other clusters differed by a maximum of two SNPs (Table 1). All clusters grouped together on a maximum-likelihood phylogeny of whole genome sequences with >95 % bootstrap support (Fig. 2). For the three samples (A1.5, B2, E1) with SNP differences between the local Nanopore-generated consensus and reference laboratory Illumina-generated consensus, both local and reference sequences clustered together on the maximum-likelihood tree with >95 % bootstrap support (Fig. 2). The six clusters of outbreak patients determined using phylogeny and SNP distances were identical to those reported as preliminary results using SNP distances alone.

On ward B, WGS demonstrated two separate bay-based clusters, linking male patients B1 and B2, and separately linking female patients B3, B4 and B5 (Table 1). Patient B3 tested negative 1 day after admission, before first testing positive 2 days after admission. It is therefore possible that patient B3 had a community-acquired IAV infection, tested negative early in infection and was placed in a bay, leading to onward transmission to bay-based contacts patients B4 and B5. In contrast, on wards A, C, D and E, WGS was consistent with clinical epidemiology, suggesting a single IAV introduction onto each ward, and transmission across different bays or between side rooms in each case (Table 1).

Both ward B and C serve cardiology and vascular patients within the same hospital building where cross-ward transmission of SARS-CoV-2 has previously occurred. Despite having only the NS segment available, patient C2 could be excluded from the ward B outbreak, with seven or more SNP differences compared to the NS segment of all ward B patients and one or fewer SNP difference compared to the NS segment of all ward C patients.

Three outbreak clusters were genetically linked by WGS to ED patients: B1 and B2 with ED96, C1, C2 and C4 with ED88, and D1, D2 and D3 with ED108 (Table 1). In each case, the genetically linked samples from ED patients were also linked in time, with a maximum of 6 days between any sample in the cluster and the linked ED sample (Fig. 2). However, there was no evidence of epidemiological linkage in space to suggest transmission.

Molecular epidemiological analysis was repeated on majority genome and HA alignments, which have been used for outbreak investigation in other studies [2, 18]. For all previously identified H3N2 and pdmH1N1 outbreak clusters, patients also clustered together with >95 % bootstrap support on maximum-likelihood phylogenies of majority genome alignments (Figs S1 and S3). However, bootstrap support for clustering E1 and E2 was lost when using the HA gene alignment alone (Fig S2). No additional ED patients clustered with outbreak patients on maximum-likelihood phylogenies of majority genome and HA gene alignments (Figs S1–S4). There was a maximum pairwise SNP distance of two between samples in outbreak clusters using a majority genome alignment, and one using the HA gene alignment (Table S4). On manual review of maximum-likelihood phylogenies of each individual segment of the genome, there was no evidence of reassortment amongst outbreak samples (Fig. S5).

### Demonstration of the utility of consensus genomes for management of individual patients

**Fig. 3.**
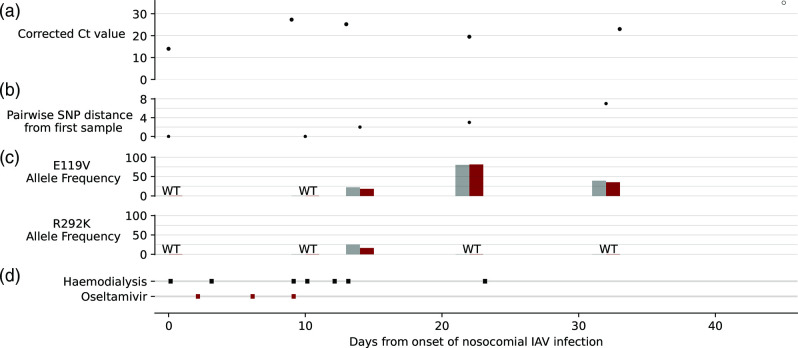
Timeline of persistent IAV infection. (**a**) AusDiagnostics nested PCR corrected Ct values, with negative result shown as a white point. (**b**) Pairwise SNP difference between the consensus genome of each sample when compared to the patient’s first sample. (**c**) Allele frequency of NA gene resistance mutations, presented as a percentage. Grey bars show Illumina data, and red bars Nanopore data. WT, wild-type. (**d**) Dates of haemodialysis and oseltamivir 30 mg IV doses.

WGS was requested for two patients for reasons other than outbreak investigation during the study period. Patient A1 remained IAV positive for more than six weeks after renal transplantation (Fig. 3a), with the final sample A1.5 differing by seven SNPs from the first sample A1.1 (Fig. 3b). He was treated with oseltamivir scheduled around haemodialysis sessions (Fig. 3d) with WGS identifying oseltamivir resistance mutations (Fig. 3c). Sample A1.4 was taken 22 days after A1.1, and 13 days after completion of oseltamivir treatment. The two consensus genomes differed by three SNPs, including a mutation in the NA gene conferring oseltamivir resistance through an A355T nucleotide substitution resulting in an E119V amino acid mutation. It was decided the patient would receive zanamivir if retreatment was required, but this was ultimately not needed. Furthermore, samples A1.3 and A1.5 demonstrated minority resistance variants in the NA gene with comparable allele frequency in both local Nanopore and reference Illumina data (Fig. 3c). Two resistant subpopulations were present in sample A1.3, with E119V present at 18 % (22 % reference Illumina data) and R292K at 16 % (25 % reference Illumina data). Reviewing the VCF files generated by IRMA for all 115 samples with an NA gene consensus sequence, no other samples had a minority variant above a frequency of 15 % at a position associated with NA inhibitor resistance, confirming the specificity of these results.

Patient R1 had a history of immunosuppression due to active low-grade B cell lymphoma and developed community-onset PCR un-typable IAV infection with a history of previous travel to Singapore. National guidance is for urgent WGS based both on the travel history to exclude avian influenza (H5N1) [22] and to inform treatment with either zanamivir (for pdmH1N1 IAV) or oseltamivir (for H3N2) [23]. WGS identified H3N2, which excluded avian influenza and informed oseltamivir treatment. This result was reported to the clinical team three working days after the clinical request.

### Demonstration of the utility of consensus genomes for timely surveillance

Of 117 samples with an available HA gene consensus sequence, 72 were H3N2 clade 3C.2a1b.2a.2, 44 pdmH1N1 clade 6B.1A.5a.2 and one sample pdmH1N1 clade 6B.1A.5a.1. Excluding patient A1’s post-treatment samples with acquired oseltamivir resistance, no NA inhibitor resistance mutations were detected in NA genes, and no mutations identified in the PA gene conferring resistance to baloxavir, a PA inhibitor.

## Discussion

Our results demonstrate the clinical utility of a 24 h Nanopore IAV WGS protocol for outbreak management, individual treatment decisions and timely surveillance. Using previously published criteria [6], WGS demonstrated two simultaneous outbreaks on a single ward and excluded cross-ward transmission. IAV WGS identified post-treatment oseltamivir resistance in a persistently infected immunocompromised patient and subtyped a sample which had failed PCR subtyping to inform treatment and exclude avian influenza. Furthermore, WGS of ED samples allowed for identification of prevalent clades and drug resistance rates which were concordant with surveillance data published by the national reference laboratory covering the same period.

The strengths of our study are the improved turnaround time and reduced hands-on time achieved by using Nanopore sequencing in an on-site laboratory when compared to previously published clinical IAV WGS studies. When used for outbreak investigation, genotypic drug resistance identification and clade assignment, the accuracy of these Nanopore-generated consensus sequences was sufficient to draw the same conclusions as when Illumina-generated reference laboratory sequences were used. Moreover, the protocol uses the same library preparation and sequencing as commercially available SARS-CoV-2 WGS kits and is therefore widely accessible to institutions with Nanopore platforms acquired during the SARS-CoV-2 pandemic.

Another strength of our study is the use of ED samples to provide a genetic background of locally circulating IAV, which demonstrated the high genetic diversity of IAV infections presenting to our healthcare institution. Nonetheless, we identified genetically linked samples from ED patients which were also linked in time with outbreak samples, but shared no identifiable contact. This highlights the need for robust clinical epidemiology when interpreting WGS results and cautions against inferring transmission events in the absence of plausible contact links.

Our study has limitations. Given the concordance of molecular and clinical epidemiology for the outbreaks in our study, which has also been reported by other investigations [18], IAV WGS had a limited impact on IPC decisions. As we were utilizing surplus clinical samples, we could not perform WGS on visitors or staff, who are not routinely tested by the IPC team. This prevented us from establishing how IAV was introduced into each ward for most outbreaks and, while there is significant diversity within the sequenced community IAV WGS samples, multiple introductions of highly similar viruses to the same ward cannot be excluded.

Although all the outbreak clusters identified in this study were within a previously derived threshold for genetic linkage, ongoing IAV WGS of hospital outbreaks is likely to identify samples with more ambiguous genetic relatedness. In particular, requests to compare samples taken more than one week apart would present a new challenge, as previous thresholds have been derived for samples taken within seven days. This issue is highlighted by the persistently infected individual in our study whose final sample, taken more than six weeks after becoming infected, differed by seven SNPs from their first sequenced sample. Clinical use of IAV WGS therefore requires careful interpretation of results, and a willingness to communicate uncertainty with IPC teams.

Although the commercial PCR assays used in this study are not quantitative, comparison to bait-capture methods, which can recover WGS for samples with Ct <37 [7], suggests that the one-step RT-PCR and Nanopore sequencing has weaker performance at lower viral loads, with only partial genomes recovered for samples with a corrected Ct >29 on the AusDiagnostics platform. The PB1 gene, which is one of the longest of the eight IAV gene segments, was prone to poor coverage, which has also been reported by other studies [17, 18]. However, using a majority genome (all segments other than PB1) did not affect phylogenetic clustering of outbreak samples, and PB1 is also not a drug target, suggesting weaker PB1 coverage does not limit the clinical utility of the protocol. In addition, homopolymer regions remain a challenge for Nanopore platforms, and a single base deletion was frequently seen in N2 NA genes in a seven-base cytosine sequence which required masking for further analysis. Homopolymer deletions and infrequent SNPs between replicates may improve on R10.4.1 Flow Cells and updated chemistry.

This study provides further evidence of the feasibility and utility of respiratory viral WGS in the clinical setting. Rapid turnaround IAV WGS can provide timely genomic surveillance, inform nosocomial outbreak management and guide individual treatment decisions in a clinically relevant timeframe. In our study IAV WGS had a clearer impact on the management of individual patients than IPC decisions, but a multi-centre study is required to confirm the generalizability of this finding. The distribution of respiratory viral WGS throughout laboratory networks has the potential to both benefit individual healthcare institutions and improve national genomic surveillance data.

## Supplementary Data

Supplementary material 1

Supplementary material 2
