# Supplementary material for: Feasibility and clinical utility of local rapid Nanopore influenza A virus whole genome sequencing for integrated outbreak management, genotypic resistance detection and timely surveillance
Source: Microb Genom. 2023 Aug 17;9(8):mgen001083. doi: 10.1099/mgen.0.001083 (PMC10483427; doi:10.1099/mgen.0.001083)
Supplement: Supplementary material 1 [file mgen-9-1083-s001.pdf]

Feasibility and clinical utility of local rapid nanopore Influenza A Virus whole genome sequencing for integrated outbreak management, genotypic resistance detection and timely surveillance.

Tom Williams*[1,3], Luke B. Snell[2,3], Chris Alder[3], Themoula Charalampous[3], Adela Alcolea-Medina[3,4], Jasveen K. Sehmi[4], Noor Al-Yaakoubi[3], Gul Humayun[3], Shahjahan Miah [5], Angie Lackenby[5], Maria Zambon[5], Rahul Batra[3], Sam Douthwaite[1], Jonathan Edgeworth[3], Gaia Nebbia*[1,2,3]

Affiliations

1. Department of Infection, Guy's & St. Thomas' NHS Foundation Trust, London, UK
2. Department of Infectious Diseases, King's College London, London, UK
3. Centre for Clinical Diagnostics & Infectious Disease Research, Guy's & St. Thomas' NHS Foundation Trust, London, UK
4. Infection Sciences, Synnovis, London, UK
5. United Kingdom Health Security Agency (UKHSA), London, United Kingdom

Supplementary Methods and Figures

Supplementary Methods – Page 3

Supplementary Figure 1 - Maximum Likelihood Phylogeny of pdmH1N1 using majority genome sequences – Page 5

Supplementary Figure 2 - Maximum Likelihood Phylogeny of pdmH1N1 using HA gene sequences – Page 7

Supplementary Figure 3 - Maximum Likelihood Phylogeny of H3N2 using majority genome sequences – Page 9

Supplementary Figure 4 - Maximum Likelihood Phylogeny of H3N2 using HA gene sequences – Page 11

Supplementary Figure 5 - Maximum Likelihood Phylogeny of individual gene segments for pdmH1N1 and H3N2 – Page 13

Supplementary methods

Consensus generation and control analysis

The FLU-minion config file was adapted as follows: the MIN_CONS_SUPPORT parameter was set to 20 to provide a consensus base call with a read depth of 20, MIN_AMBIG set to 0.5 to prevent mixed base calls, and ASSEM_PROG set to MINIMAP2.

There was a maximum pairwise SNP distance of 1 between nine H3N2 control replicates (seven identical replicates, two replicates sharing one SNP in the HA gene) and eight H1N1 control replicates (seven identical replicates, one replicate with one SNP in the HA gene). All eight H3N2 control whole genome sequences had a one base deletion in a seven-base cytosine homopolymer in the NA gene. This deletion was therefore considered a nanopore sequencing error, and positions 131 to 137 in the N2 NA gene alignment masked in further analysis. No consensus bases were called in any negative control sample. The in-run lambda phage DNA control was used to estimate barcode misassignment. Across all sequencing runs, the median number of reads mapping to the lambda genome (NC_001416.1) from IAV barcodes was 0, and only one IAV barcode had >5 reads mapping to the lambda genome, suggesting that barcode misassignment occurred at very low levels.

Comparison with Illumina-generated genomes

22 samples were also sequenced by the reference laboratory using an Illumina pipeline and consensus sequences compared (Supplementary Table 1). At the reference laboratory, multisegment reverse transcription-PCR (M-RTPCR)[1] was used to amplify influenza virus-specific segments. Reverse-transcription and amplification was performed using one-step RT-PCR system with Superscript III and Platinum Taq HiFi polymerase (Life Technologies). After preparing the amplicons using Nextera XT library preparation kit (Illumina), samples were sequencing on an Illumina NextSeq producing 150bp-paired end reads. The sequence data generated was processed using BWA-MEM to map the reads to appropriate reference sequences. Samtools was used to post-process the reference assembly and an in-house C++ program (QuasiBam) used to quality filter, trim and generate two outputs: a consensus sequence for the

influenza genomes and a table with the frequency of each nucleotide and depth at every position. Positions in the consensus genome showing greater than 20% variance were assigned an ambiguity code.

For the 18 samples with whole genome sequences available from both Illumina and nanopore methods, 15 samples had no SNP differences between the consensus sequences generated by the different methods. The other three samples had one or two SNP differences between the consensus sequences in a single polymerase gene: sample B2 had one SNP in the PB2 gene (position 288), sample A1.5 had two SNPs in the PB2 gene (positions 1690 and 2250), and sample E2 had two SNPs in the PA gene (positions 1691 and 1697). The SNP positions were all covered at a depth >100 in the nanopore data. Partial genomes with at least one segment for comparison were available for comparison for another four samples and had no SNP differences between the Illumina and nanopore consensus sequences.

1 Zhou B, Donnelly ME, Scholes DT, *et al.* Single-Reaction Genomic Amplification Accelerates Sequencing and Vaccine Production for Classical and Swine Origin Human Influenza A Viruses. *J Virol* 2009; **83**: 10309–13.

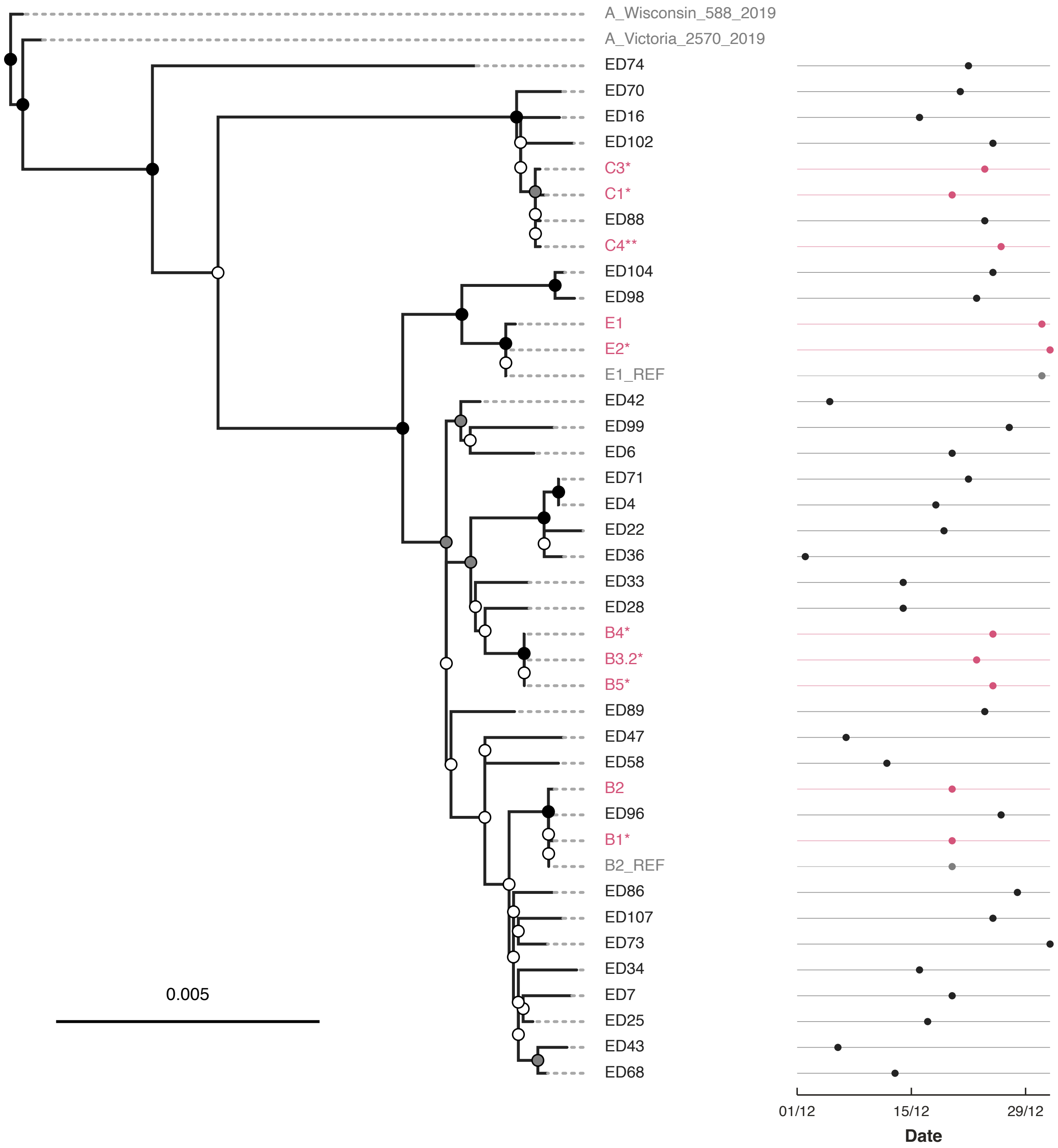

Supplementary Figure 1 - Maximum Likelihood Phylogeny of pdmH1N1 using majority genome sequences – Alphanumeric names refer to patient location (for example A for ward A, ED for Emergency Department) followed by a patient number. Patients with multiple samples are labelled using a numerical suffix, for example A1.1 for the first sample and A1.2 for the second sample. Samples from outbreak patients and clinical requests are highlighted in red. *indicates samples with no SNP differences to a reference laboratory complete genome sequence, **indicates samples with no SNP differences to a reference

laboratory partial genome sequence. Nodes colour represents bootstrap support, with black nodes indicating 100% support, grey 95-99% support, and white <95% support. Outgroups are the Northern Hemisphere Winter Season 2022/2023 vaccine strains A/Victoria/2570/2019 (EPI_ISL_417210) and A/Wisconsin/588/2019 (EPI_ISL_404460).

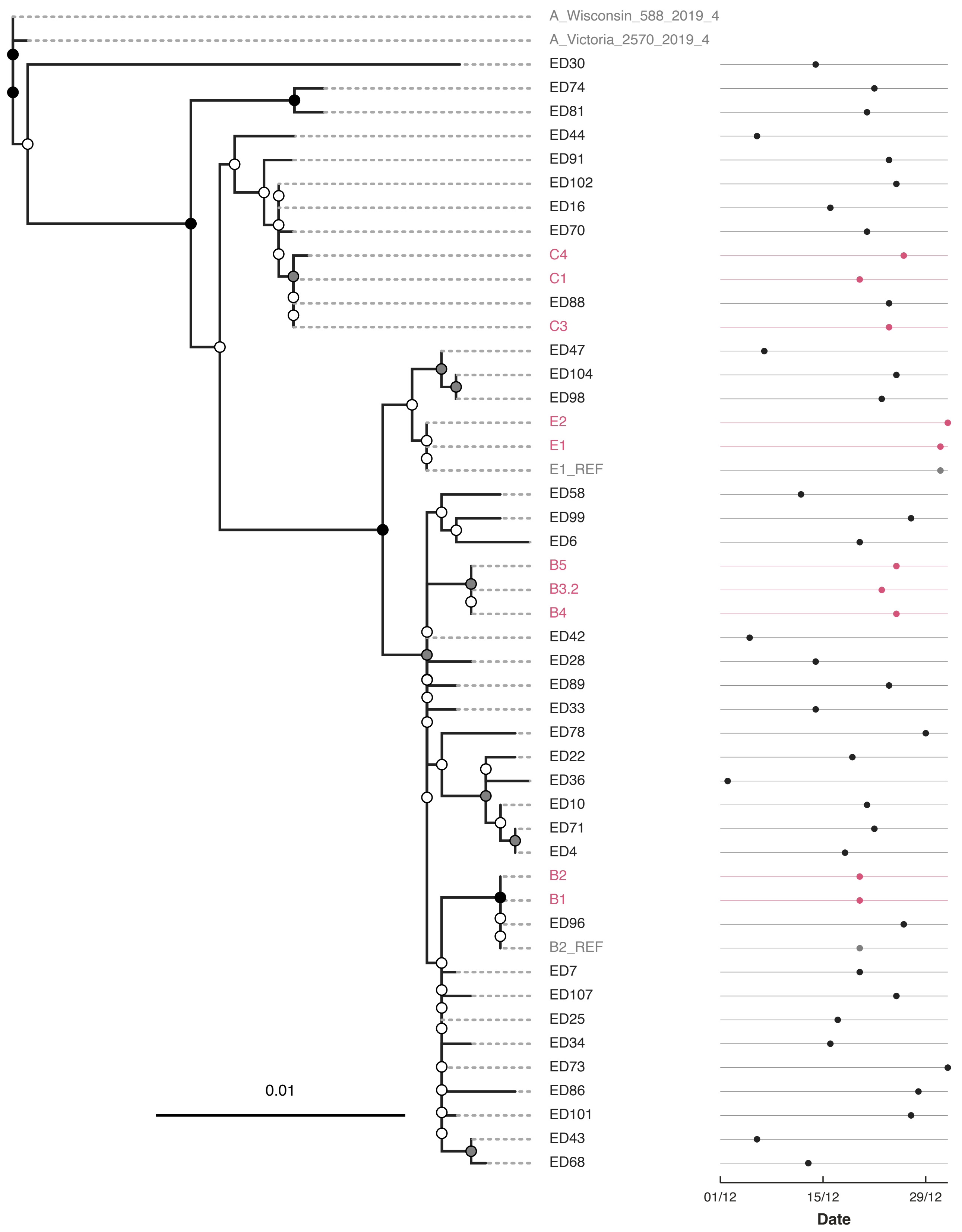

Supplementary Figure 2 - Maximum Likelihood Phylogeny of pdmH1N1 using HA gene sequences – Alphanumeric names refer to patient location (for example A for ward A, ED for Emergency Department) followed by a patient number. Patients with multiple samples are labelled using a numerical suffix, for

example A1.1 for the first sample and A1.2 for the second sample. Samples from outbreak patients and clinical requests are highlighted in red. Nodes colour represents bootstrap support, with black nodes indicating 100% support, grey 95-99% support, and white <95% support. Outgroups are the Northern Hemisphere Winter Season 2022/2023 vaccine strains A/Victoria/2570/2019 (EPI_ISL_417210) and A/Wisconsin/588/2019 (EPI_ISL_404460).

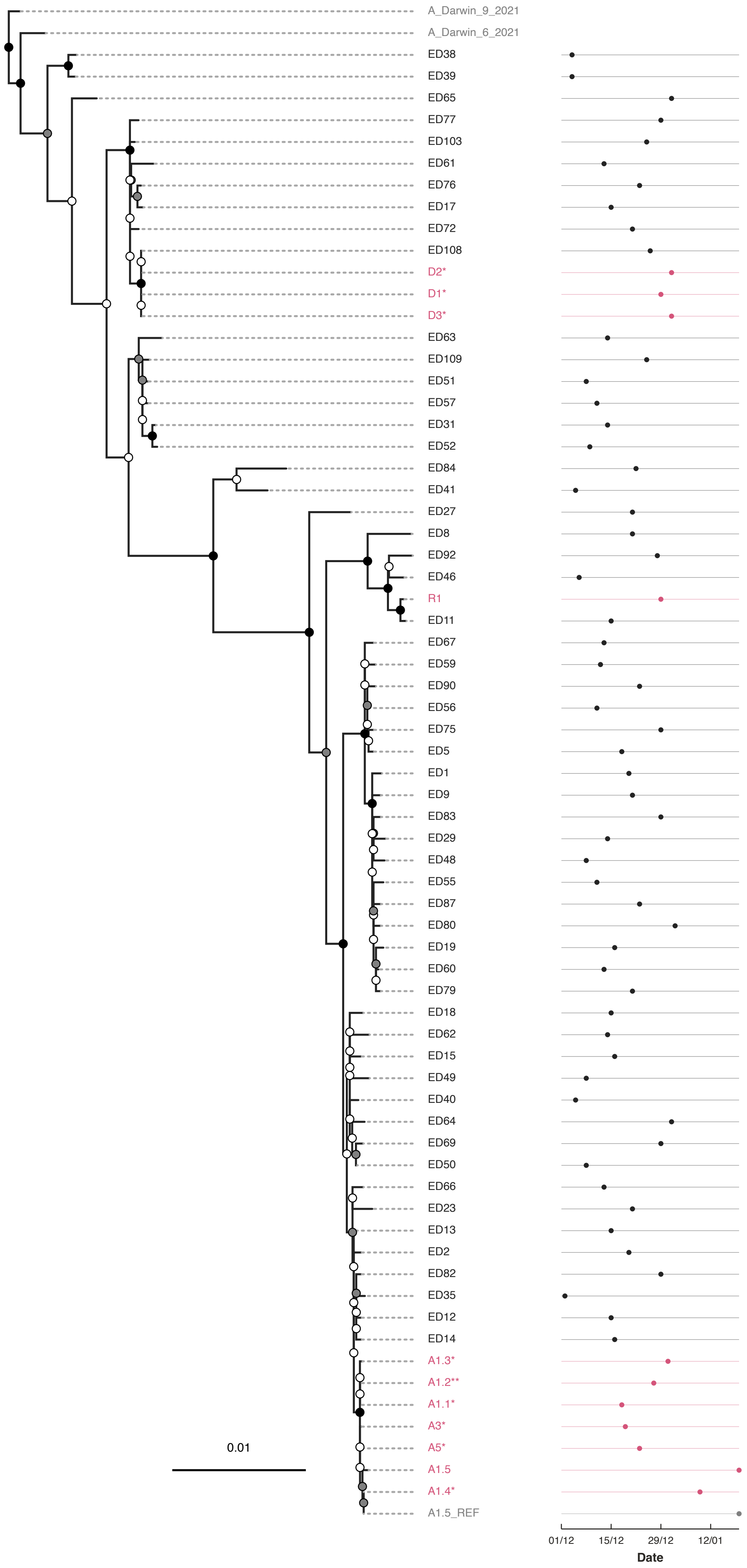
A_Darwin_9_2021
A_Darwin_6_2021
ED38
ED39
ED65
ED77
ED103
ED61
ED76
ED17
ED72
ED108
D2*
D1*
D3*
ED63
ED109
ED51
ED57
ED31
ED52
ED84
ED41
ED27
ED8
ED92
ED46
R1
ED11
ED67
ED59
ED90
ED56
ED75
ED5
ED1
ED9
ED83
ED29
ED48
ED55
ED87
ED80
ED19
ED60
ED79
ED18
ED62
ED15
ED49
ED40
ED64
ED69
ED50
ED66
ED23
ED13
ED2
ED82
ED35
ED12
ED14
A1.3*
A1.2**
A1.1*
A3*
A5*
A1.5
A1.4*
A1.5_REF
0.01
01/12
15/12
29/12
12/01
Date

Supplementary Figure 3 - Maximum Likelihood Phylogeny of H3N2 using majority genome sequences – Alphanumeric names refer to patient location (for example A for ward A, ED for Emergency Department) followed by a patient number. Patients with multiple samples are labelled using a numerical suffix, for example A1.1 for the first sample and A1.2 for the second sample. Samples from outbreak patients and clinical requests are highlighted in red. *indicates samples with no SNP differences to a reference laboratory complete genome sequence, **indicates samples with no SNP differences to a reference laboratory partial genome sequence. Nodes colour represents bootstrap support, with black nodes indicating 100% support, grey 95-99% support, and white <95% support. Outgroups are the Northern Hemisphere Winter Season 2022/2023 vaccine strains A/Darwin/9/2021 (EPI_ISL_2233240) and A/Darwin/6/2021 (EPI_ISL_2233238) for H3N2.

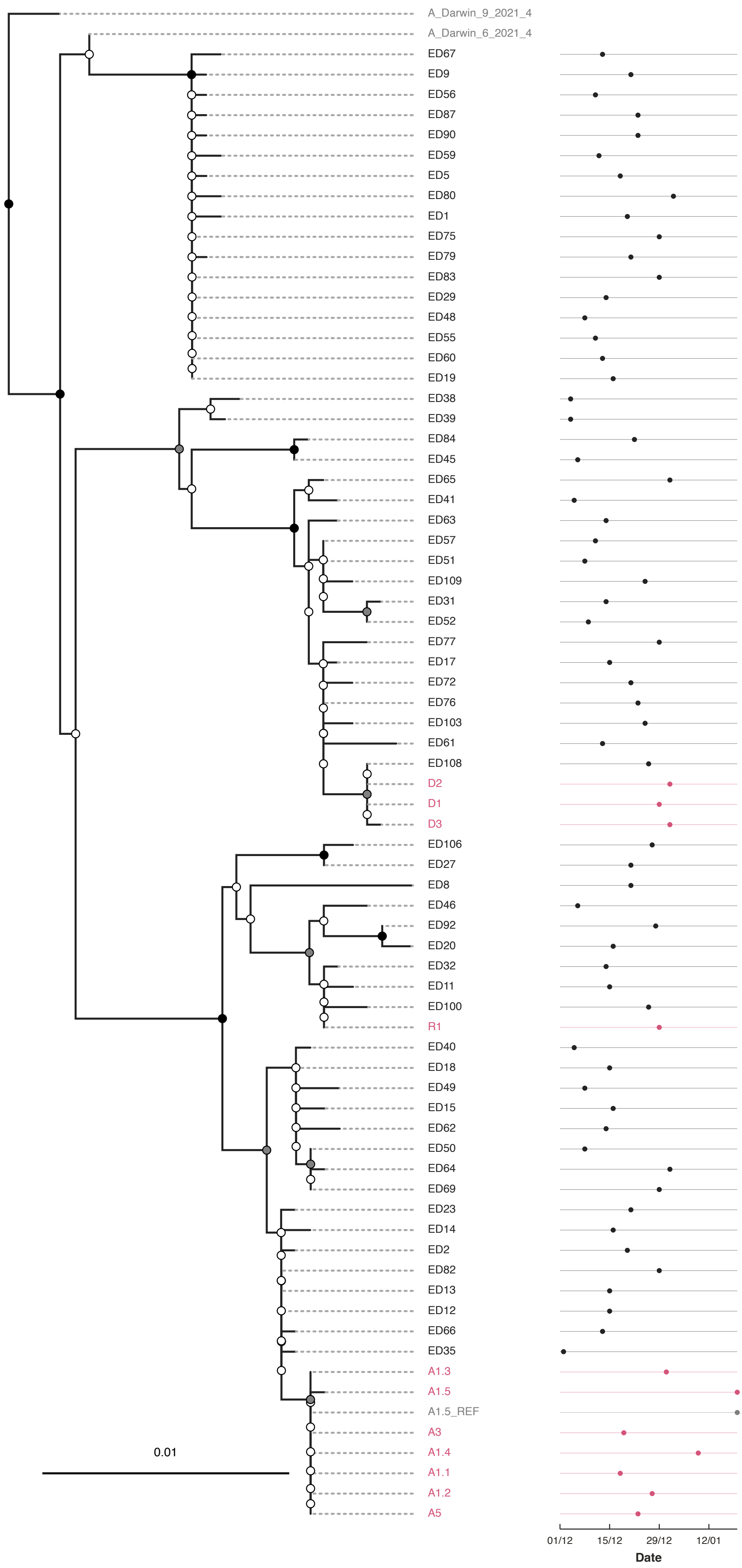

A_Darwin_9_2021_4
A_Darwin_6_2021_4
ED67
ED9
ED56
ED87
ED90
ED59
ED5
ED80
ED1
ED75
ED79
ED83
ED29
ED48
ED55
ED60
ED19
ED38
ED39
ED84
ED45
ED65
ED41
ED63
ED57
ED51
ED109
ED31
ED52
ED77
ED17
ED72
ED76
ED103
ED61
ED108
D2
D1
D3
ED106
ED27
ED8
ED46
ED92
ED20
ED32
ED11
ED100
R1
ED40
ED18
ED49
ED15
ED62
ED50
ED64
ED69
ED23
ED14
ED2
ED82
ED13
ED12
ED66
ED35
A1.3
A1.5
A1.5_REF
A3
A1.4
A1.1
A1.2
A5
0.01
01/12
15/12
29/12
12/01
Date

Supplementary Figure 4 - Maximum Likelihood Phylogeny of H3N2 using HA gene sequences – Alphanumeric names refer to patient location (for example A for ward A, ED for Emergency Department) followed by a patient number. Patients with multiple samples are labelled using a numerical suffix, for example A1.1 for the first sample and A1.2 for the second sample. Samples from outbreak patients and clinical requests are highlighted in red. Nodes colour represents bootstrap support, with black nodes indicating 100% support, grey 95-99% support, and white <95% support. Outgroups are the Northern Hemisphere Winter Season 2022/2023 vaccine strains A/Darwin/9/2021 (EPI_ISL_2233240) and A/Darwin/6/2021 (EPI_ISL_2233238) for H3N2.

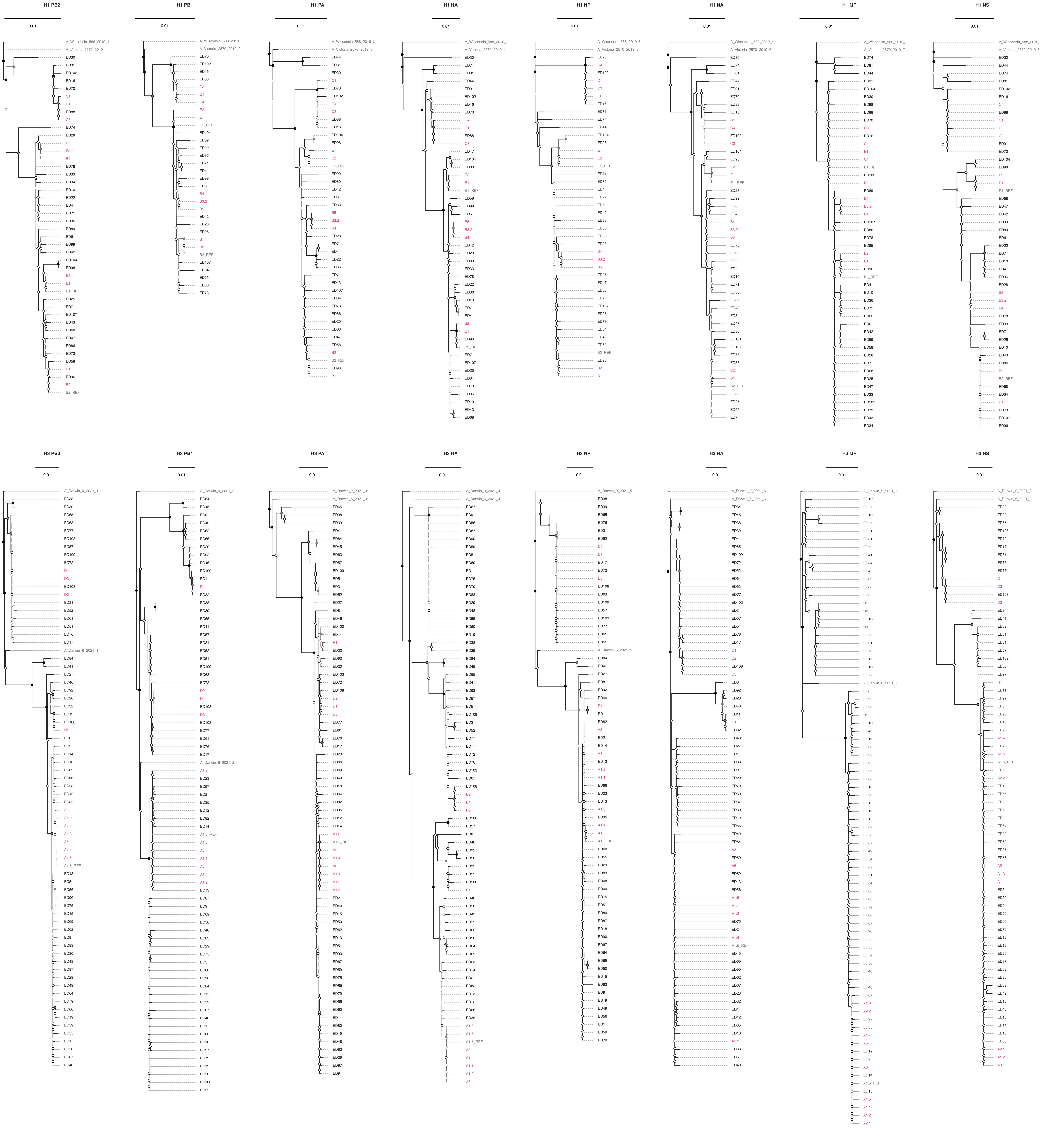
H1 PB2
H1 PB1
H1 PA
H1 HA
H1 NP
H1 NA
H1 MP
H1 NS
H3 PB2
H3 PB1
H3 PA
H3 HA
H3 NP
H3 NA
H3 MP
H3 NS
0.01

Supplementary Figure 5 - Maximum Likelihood Phylogeny of individual gene segments for pdmH1N1 and H3N2 – Alphanumeric names refer to patient location (for example A for ward A, ED for Emergency Department) followed by a patient number. Patients with multiple samples are labelled using a numerical suffix, for example A1.1 for the first sample and A1.2 for the second sample. Samples from outbreak patients and clinical requests are highlighted in red. Nodes colour represents bootstrap support, with black nodes indicating 100% support, grey 95-99% support, and white <95% support. Outgroups are the Northern Hemisphere Winter Season 2022/2023 vaccine strains A/Victoria/2570/2019 (EPI_ISL_417210) and A/Wisconsin/588/2019 (EPI_ISL_404460) for pdmH1N1 and A/Darwin/9/2021 (EPI_ISL_2233240) and A/Darwin/6/2021 (EPI_ISL_2233238) for H3N2.
